# Measures to sustain endangered languages: A bilingual competition model with sliding mode control

**DOI:** 10.1371/journal.pone.0287850

**Published:** 2023-06-29

**Authors:** Ya Gao, WenQi Liu

**Affiliations:** 1 Faculty of Science, Kunming University of Science and Technology, Kunming, China; 2 Data Science Research Center, Kunming University of Science and Technology, Kunming, China; Tallinn University: Tallinna Ulikool, ESTONIA

## Abstract

There are thousands of languages in the world, many of which are in danger of extinction due to language competition and evolution. Language is an aspect of culture, the rise, and fall of a language directly affects its corresponding culture. To preserve languages and prevent their mass extinction, it is crucial to develop a mathematical model of language coexistence. In this paper, we use a qualitative theory of ordinary differential equations to analyze the bilingual competition model, and obtain the trivial and non-trivial solutions of the bilingual competition model without sliding mode control, then analyze the stability of solutions and prove that solutions of the model have positive invariance. In addition, to maintain linguistic diversity and prevent mass extinction of languages, we propose a novel bilingual competition model with sliding control. The bilingual competition model is analyzed by proposing a sliding control policy to obtain a pseudo-equilibrium point. Meanwhile, numerical simulations clearly illustrate the effectiveness of the sliding mode control strategy. The results show that the likelihood of successful language coexistence can be increased by changing the status of languages and the value of monolingual-bilingual interaction, provides theoretical analysis for the development of policies to prevent language extinction.

## Introduction

There are about 6000 languages spoken in the world, and different languages are the product of the cultural evolution of different peoples in different environments and are a valuable part of human culture. The study of language evolution has an important role in understanding the diversity of human cultures. Therefore, it is a good approach to study the dynamics of language using evolutionary dynamics methods. Szathmáry and Smith [1]identified the emergence of language as one of the major transitions in the evolutionary process. Language provides opportunities for cultural evolution, which is a focus of evolutionary biology [2, 3].

The explosive expansion of some languages in recent decades has left at least half of the world’s languages in an endangered state [4]. Globally, more than 750 languages have become extinct, and many others have only a few speakers [5]. Based on language extinction trends, some of them are mentioned in [6–8], and references therein. The causes of language endangerment are complex and are influenced by the evolution of the internal structure of the language and changes in the external environment of the language. These factors constitute a complex system that affects languages. Therefore, to save endangered languages, it is necessary to conduct an in-depth study of the mechanisms and dynamics of language transmission, competition, and regulation to reveal the causes of language decline, extinction, and coexistence, and then intervene in endangered language preservation and policies.

Research on language evolution has language origins and language competition. The former is usually conducted in an interdisciplinary manner with relatively obvious intersectional features in linguistics, evolutionary biology, neurological and functional brain sciences, and computer science [9–11]. On the other hand, studies on language competition are usually considered more quantitative because the various models in language competition studies allow for quantitative analysis and assessment of language competition trends. This paper focuses on the latter.

In [12], Abrams-Strogatz use mathematical models to describe language competition, who proposed two competing language groups in a stable population, with changes between language groups determined by the social status of the language and predicted the inevitable extinction of one of the languages, with a theoretical model that matched the actual data. This article is considered a seminal work in the study of language competition and extinction using a mathematical modeling approach and has influenced most of the research literature published on this topic since then. In [13], Mira and Paredes et al. extended the Abrams-Strogatz model, which allows the use of the presence of bilingual and monolingual speakers and includes a parameter indicating bilingual similarity, showing through data for Spanish and Galician that bilingual groups can coexist stably if the competing languages are sufficiently similar. In [14], Mira et al. demonstrated the importance of stable bilingual groups for the coexistence of the two languages. [15], Espinar and Seoane et al. explain the results of other analyses of the [14] model. Gong and Zhang [16] proposed a new language competition model based on the Lotka-Volterra [17] evolutionary biology competition model, using census data and Geographic information data to make the parameters of the language competition model estimable and using examples to verify the feasibility and correctness of the theory and model. Prochazka and Vogl [18] introduced that language shift can be monitored on a large scale through the reaction-diffusion equations and proposed a language dynamics model based on the principles of agent-based modeling. The model can track the dynamics of language in space and time, with results that are robustly compatible with empirical data.

Regarding the control of endangered languages, in [19], the control of biology is applied to the control of languages, and Nie et al. establish an optimal control problem to protect endangered languages. In [20], Nie et al. propose a new model of competition between two languages with bilingualism and linguistic similarity, in which a state-dependent impulse control strategy is introduced. The results show that the ratio of two competing languages can be kept at a reasonable level in almost any case and provide a theoretical basis for control measures to protect endangered languages.

The above-mentioned studies have investigated language transmission and control from different perspectives. However, most of the studies are based on the A-S model for expansion. Since the A-S model assumes that a language can legacy its own language to another language this is not realistic. In practice, language transition is required from a bilingual state to another language. In [21], based on the Abrams-Strogatz model, Minett and Wang proposed a different way to model language transmission by introducing vertical (parental) and horizontal (peer) transmission of language, and a partial analysis of the model. However, Minett and Wang only partially simulated their model and did not perform an algebraic analysis of the other parametric forms of the model. In particular, Abrams-Strogatz [12], Mira and Paredes [13], Mira et al. [14] found that this parameter is roughly constant across cultures for historical reasons, with *a* = 1.31±0.25. Thus, it is more realistic to study the dynamic behavior for the parameter *a* ≥ 1.

To address the above issues, a more in-depth study of language propagation competition and language control mechanisms is conducted. In this paper, Minett-Wang’s model is extended to introduce population interaction values, considering language transmission characteristics. A comprehensive algebraic analysis of the proposed bilingual competitive model is performed using the qualitative theory of ordinary differential equations, and the dynamical behavior of the Minett-Wang model with parameter *a* ≥ 1 is extended to obtain the nontrivial and non-trivial solutions of the model without sliding mode control. Then we analyze the stability of the solutions to prove that the solutions of the model have positive invariance. In addition, to maintain language diversity, prevent language mass extinction, and make language coexistence possible, we propose a novel bilingual competition model with sliding control. A sliding control strategy is used to obtain the pseudo-equilibrium point of the model and the validity of the model is verified by numerical experiments with sliding mode control. The findings suggest that the possibility of language coexistence can be increased by changing the status of languages and the value of mono-bilingual interactions, providing theoretical analysis and guidance for the development of policies to prevent language extinction.

The rest of the paper is structured as follows. The bilingual competition model without sliding mode control is presented in Section 2, and a qualitative theoretical analysis of the bilingual competition model without sliding mode control is given in Section 3. In Section 4, numerical simulations of the bilingual competition model without sliding mode control are given. Section 5 proposes a new bilingual competition model with sliding. The conclusion is presented in Section 6.

## The bilingual competition model without sliding mode control

Taking into account the characteristics of languages, language transfer depends on the interaction of population density is introduced in [22–26]. We extend the Minett and Wang model by introducing the population interaction value into the model. The model is described by the following differential equation:

$$\begin{array}{c} \left\{ \begin{array}{c} \frac{dx_{A}}{dt}=\mu wP_{w\cdot x_{A}}-\left(1-\mu \right)x_{A}P_{x_{A}\cdot w},\\ \frac{dx_{B}}{dt}=\mu wP_{w\cdot x_{B}}-\left(1-\mu \right)x_{B}P_{x_{B}\cdot w}. \end{array}\right. \end{array}$$ (1)

Where *X*_*A*_(*t*), *X*_*B*_(*t*) represents the number of people who speak only monolingual *A*, *B* at time *t*, *W* represents the number of people who are bilinguals (i.e. speak both languages) at time *t*, and the total population size over time is denoted *N*(*t*) = *X*_*A*_(*t*) + *X*_*B*_(*t*) + *W*(*t*). The population proportions of monolingual speakers of *A*, *B* and bilinguals *w* are denoted *x*_*A*_(*t*) = *X*_*A*_(*t*)/*N*(*t*), *x*_*B*_(*t*) = *X*_*B*_(*t*)/*N*(*t*), *w*(*t*) = *W*(*t*)/*N*(*t*) espectively, satisfy *x*_*A*_(*t*) + *x*_*B*_(*t*) + *w*(*t*) = 1. *μ* represents mortality rate. The parameter $P_{w\cdot x_{A}},P_{w\cdot x_{B}}$ represents the transfer rate from bilinguals to monolingual speakers of *A*, *B*, which is determined by the status of language *A*, *B* and the proportion of monolingual speakers of *A*, *B*, with the other parameters denoted $P_{x_{A}\cdot w},P_{x_{B}\cdot w}$ by similar symbols and defined in the following:

$$\begin{array}{c} \begin{array}{c} P_{w\cdot x_{A}}=I_{w\cdot x_{A}}s_{x_{A}}x_{A}^{a},P_{x_{A}\cdot w}=I_{x_{A}\cdot w}s_{x_{B}}x_{B}^{a},\\ P_{w\cdot x_{B}}=I_{w\cdot x_{B}}s_{x_{B}}x_{B}^{a},P_{x_{B}\cdot w}=I_{x_{B}\cdot w}s_{x_{A}}x_{A}^{a}. \end{array} \end{array}$$ (2)

Where $s_{x_{A}},s_{x_{B}}$ indicates the status of language *A*, *B*, $I_{w\cdot x_{A}},I_{w\cdot x_{B}}$ denotes the interaction value from bilinguals to monolingual speakers of *A*, *B*, $I_{x_{A}\cdot w},I_{x_{B}\cdot w}$ denotes the interaction value from monolingual speakers of *A*, *B* to bilinguals. The parameter *a* indicates how the attractiveness of monolingual *A*, *B* corresponds to the proportion of the number of people speaking the language *A*, *B*. When *a* = 1, it means that the attractiveness of the monolingual *A*, *B* increases linearly with the proportion of speakers. When *a* = 2, it means that the attractiveness of the monolingual *A*, *B* increases superlinearly with the proportion of speakers. When *a* = 0.5, it means that the attractiveness of the monolingual *A*, *B* increases sublinearly with the proportion of speakers.

Bringing the parameters formula (2) into the model (1), we get the following reduced model:

$$\begin{array}{c} \left\{ \begin{array}{c} \frac{dx_{A}}{dt}=\mu wI_{w\cdot x_{A}}s_{x_{A}}x_{A}^{a}-\left(1-\mu \right)x_{A}I_{x_{A}\cdot w}s_{x_{B}}x_{B}^{a},\\ \frac{dx_{B}}{dt}=\mu wI_{w\cdot x_{B}}s_{x_{B}}x_{B}^{a}-\left(1-\mu \right)x_{B}I_{x_{B}\cdot w}s_{x_{A}}x_{A}^{a}. \end{array}\right. \end{array}$$ (3)

**Fig 1:**
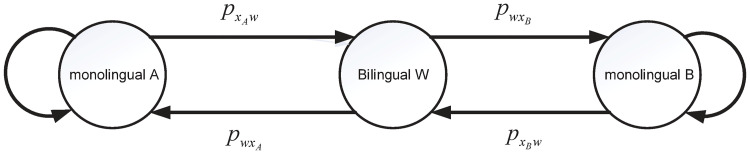
The bilingual competition model without sliding mode control transfer rate. The transfer rate from bilingual to monolingual A(B) is $P_{w\cdot x_{A}}\left(P_{w\cdot x_{B}}\right)$, and the probability of transferring monolingual A(B) to bilingual is $P_{x_{A}\cdot w}\left(P_{x_{B}\cdot w}\right)$.

The parameters have the same meaning as before. The bilingual competition model without sliding mode control is shown in Fig 1. The assumptions of the model are: the population size is relatively stable regardless of population growth; the population has a single social structure and individuals are connected and interact with each other. Although this is a strong assumption, it does exist in real life, e.g., in the border areas of Xishuangbanna and Lincang in Yunnan, China, where mutual influence and communication are relatively frequent.

To qualitatively analyze model (3), Let $\mu I_{w\cdot x_{A}}s_{x_{A}}=a_{1},\left(1-\mu \right)I_{x_{A}\cdot w}s_{x_{B}}=a_{2}$, $\mu I_{w\cdot x_{B}}s_{x_{B}}=a_{3},\left(1-\mu \right)I_{x_{B}\cdot w}s_{x_{A}}=a_{4}$, Also, them being positive can be summed up by *μ* < 1, i.e. the whole population cannot die in a single time step. we reduce Eq (3) to Eq (4).

$$\begin{array}{c} \left\{ \begin{array}{c} \frac{dx_{A}}{dt}=a_{1}\left(1-x_{A}-x_{B}\right)x_{A}^{a}-a_{2}x_{A}x_{B}^{a}\\ \frac{dx_{B}}{dt}=a_{3}\left(1-x_{A}-x_{B}\right)x_{B}^{a}-a_{4}x_{B}x_{A}^{a}. \end{array}\right. \end{array}$$ (4)

Where *x*_*A*_, *x*_*B*_ denotes the proportion of monolingual *A*, *B* populations to the total population, respectively. The parameter *a* indicates how the attractiveness of monolingual *A*, *B* corresponds to the proportion of the number of people speaking the language *A*, *B*. In particular, the researchers found that this parameter is roughly constant across cultures for historical reasons, with *a* = 1.31 ± 0.25 [12–14]. Therefore, we should study the behavior of the model (4) for *a* ≥ 1, which is a more interesting generalization.

## Qualitative analysis for model (4)

### Adaptability analysis

**Fig 2:**
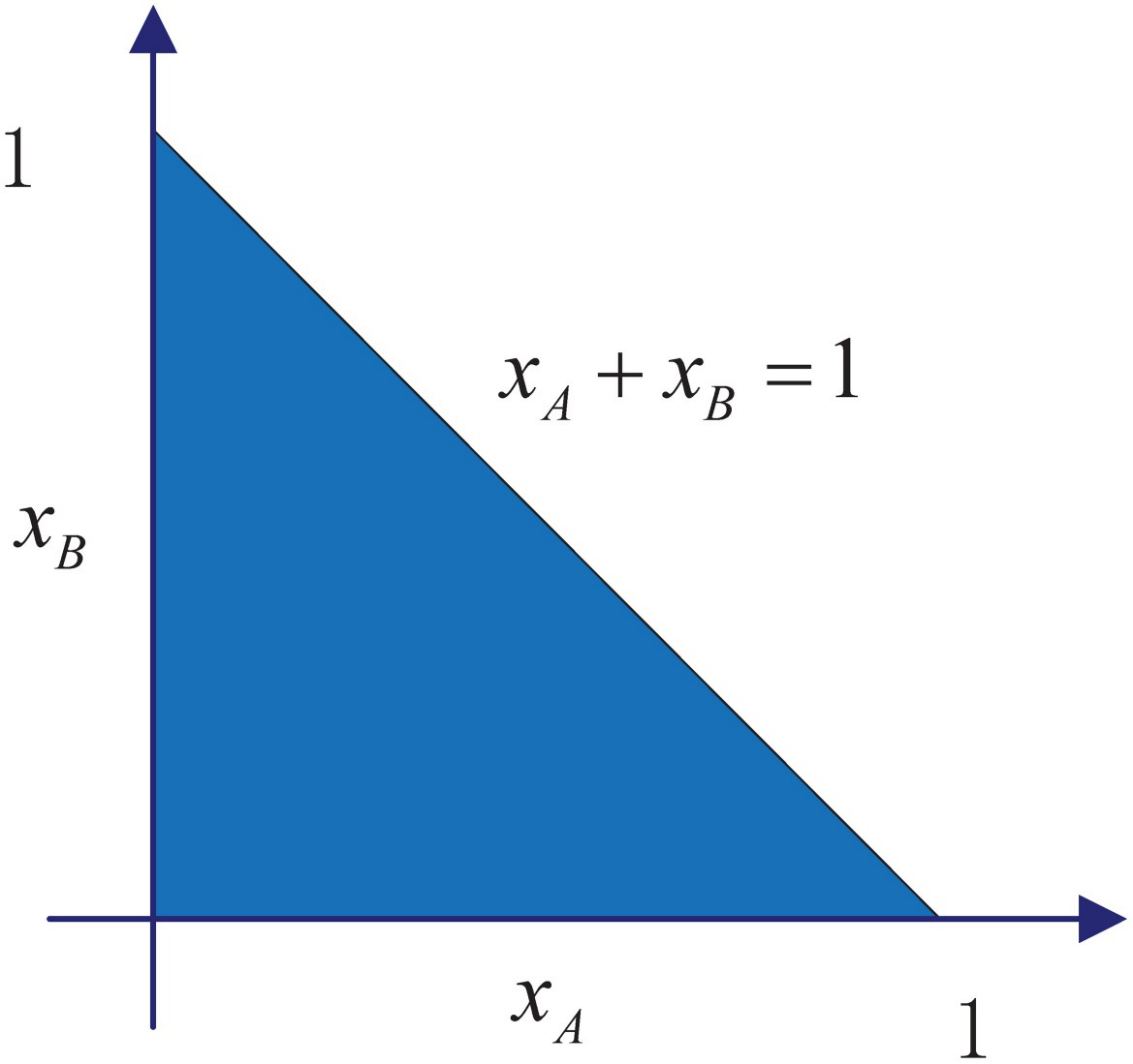
Phase plane of monolingual groups *x*_*A*_ and *x*_*B*_ in Ω. Ω = {(*x*_*A*_, *x*_*B*_):*x*_*A*_ ⩾ 0, *x*_*B*_ ⩾ 0, *x*_*A*_ + *x*_*B*_ ⩽ 1}.

We note that all possible distributions between monolingual groups *A* and monolingual groups *B* can be represented in the *x*_*A*_ − *x*_*B*_ space. Considering the context of the model, we consider model (4) only to the extent that it makes sense Ω = {(*x*_*A*_, *x*_*B*_):*x*_*A*_ ⩾ 0, *x*_*B*_ ⩾ 0, *x*_*A*_ + *x*_*B*_ ⩽ 1}, as shown in Fig 2.

The following theorem is about the non-negativity of solution of model (4).

**Theorem 1**. *The region* Ω *is positively invariant*.

*Proof*. The vector field defined by the model (4) flows inside the region Ω.

 1. If *x*_*A*_ = 0, *x*_*B*_ = [0, 1], we have

$\frac{dx_{A}}{dt}{|}_{\left(0,x_{B}\right)}=0$,

$\frac{dx_{B}}{dt}{|}_{\left(0,x_{B}\right)}=a_{3}\left(1-x_{B}\right)x_{B}^{a}⩾0.$

 When $0⩽x_{B}⩽1,\frac{dx_{B}}{dt}{|}_{\left(0,x_{B}\right)}⩾0$, the second inequality implies that the vector field flow to the region Ω.

 2. If *x*_*B*_ = 0, *x*_*A*_ = [0, 1], we have

$\frac{dx_{A}}{dt}{|}_{\left(x_{A},0\right)}=a_{1}\left(1-x_{A}\right)x_{A}^{a}⩾0$,

$\frac{dx_{B}}{dt}{|}_{\left(x_{A},0\right)}=0.$

 When $0⩽x_{A}⩽1,\frac{dx_{B}}{dt}{|}_{\left(x_{A},0\right)}⩾0$, the second inequality implies that the vector field flow to the region Ω.

 3. If *x*_*B*_ = 1 − *x*_*A*_, we have

$\frac{dx_{A}}{dt}{|}_{\left(x_{A},1-x_{A}\right)}=-a_{2}x_{A}{\left(1-x_{A}\right)}^{a}$,

$\frac{dx_{B}}{dt}{|}_{\left(x_{A},1-x_{A}\right)}=-a_{4}x_{A}^{a}\left(1-x_{A}\right).$

 In this case, the vector field flows into the region when and only when $-\frac{dx_{A}}{dt}{{|}_{\left(x_{A},1-x_{A}\right)}-\frac{dx_{B}}{dt}|}_{\left(x_{A},1-x_{A}\right)}⩾0$. Since (−1, 1) is the normal vector of the line *x*_*B*_ = 1 − *x*_*A*_, when *x*_*A*_ ∈ (0, 1) vector field flows inside the region Ω. In summary, the vector field defined by the model (4) flows inside the region Ω.

### Existence analysis

To find equilibria points, let the right-hand side of Eq (4) be zero to obtain Eq (5):

$$\begin{array}{c} \left\{ \begin{array}{c} a_{1}\left(1-x_{A}-x_{B}\right)x_{A}^{a}-a_{2}x_{A}x_{B}^{a}=0,\\ a_{3}\left(1-x_{A}-x_{B}\right)x_{B}^{a}-a_{4}x_{B}x_{A}^{a}=0. \end{array}\right. \end{array}$$ (5)

In Ω, we observe that for any value of the parameter *a*_1_, *a*_2_, *a*_3_, *a*_4_, equilibria points that can be detected by a simple inspection of the model (4) are *E*_0_(0, 0), *E*_1_(0, 1), *E*_2_(1, 0). We will term them trivial fixed points.

We analyze the equilibrium point *E*_0_(0, 0), which implies the absence of monolingual group *A* and monolingual group *B*. Individuals in groups in the community are bilinguals. As time evolves, because of socio-economic factors and personal factors. It is possible that there are no monolingual groups in the community and that all individuals in groups are bilinguals. It is only the degree of proficiency in the two languages that varies, and we do not discuss this situation here. Bilingualism is more common and the most important social phenomenon among Yunnan’s minority groups. For example, the Mongols living in the Mongolian township of Xingmeng are bilingual in Chinese in addition to their own Ka Zhuo language.

We analyze the equilibrium point *E*_1_(0, 1), *E*_2_(1, 0), which means that there are only monolingual groups present in the community. The demise of the monolingual group because of language competition indicates a situation in which the language becomes homogeneous and no longer diverse. Language extinction is irreversible, so we need to prevent this from happening. This phenomenon is also found in Yunnan’s minority groups, where the native language has disappeared and been converted to Chinese, such as the Shui ethnic group living in Fuyuan, Qujing, who use Chinese and whose native language has largely disappeared.

Depending on different values of the mentioned parameters we shall find more equilibria points. In the region{(*x*_*A*_, *x*_*B*_):*x*_*A*_ > 0, *x*_*B*_ > 0, *x*_*A*_ + *x*_*B*_ < 1}, suppose that there exists an equilibrium point $E_{3}\left(x_{A}^{*},x_{B}^{*}\right)$, which satisfies Eq (5), then $a_{1}\left(1-x_{A}^{*}-x_{B}^{*}\right){\left(x_{A}^{*}\right)}^{a}-a_{2}x_{A}{\left(x_{B}^{*}\right)}^{a}=0,a_{3}\left(1-x_{A}^{*}-x_{B}^{*}\right){\left(x_{B}^{*}\right)}^{a}-a_{4}x_{B}{\left(x_{A}^{*}\right)}^{a}=0$. The parameter *a* ≥ 1, *a*_1_ > 0, *a*_2_ > 0, *a*_3_ > 0, *a*_4_ > 0, by calculation, we get $x_{A}^{*}=\frac{a_{1}{\left(\frac{a_{2}a_{3}}{a_{1}a_{4}}\right)}^{\frac{a+1}{2a-1}}}{a_{1}{\left(\frac{a_{2}a_{3}}{a_{1}a_{4}}\right)}^{\frac{a+1}{2a-1}}+a_{1}{\left(\frac{a_{2}a_{3}}{a_{1}a_{4}}\right)}^{\frac{a}{2a-1}}+a_{2}{\left(\frac{a_{2}a_{3}}{a_{1}a_{4}}\right)}^{\frac{1}{2a-1}}}$, $x_{B}^{*}=\frac{a_{1}{\left(\frac{a_{2}a_{3}}{a_{1}a_{4}}\right)}^{\frac{a}{2a-1}}}{a_{1}{\left(\frac{a_{2}a_{3}}{a_{1}a_{4}}\right)}^{\frac{a+1}{2a-1}}+a_{1}{\left(\frac{a_{2}a_{3}}{a_{1}a_{4}}\right)}^{\frac{a}{2a-1}}+a_{2}{\left(\frac{a_{2}a_{3}}{a_{1}a_{4}}\right)}^{\frac{1}{2a-1}}}$.

**Fig 3:**
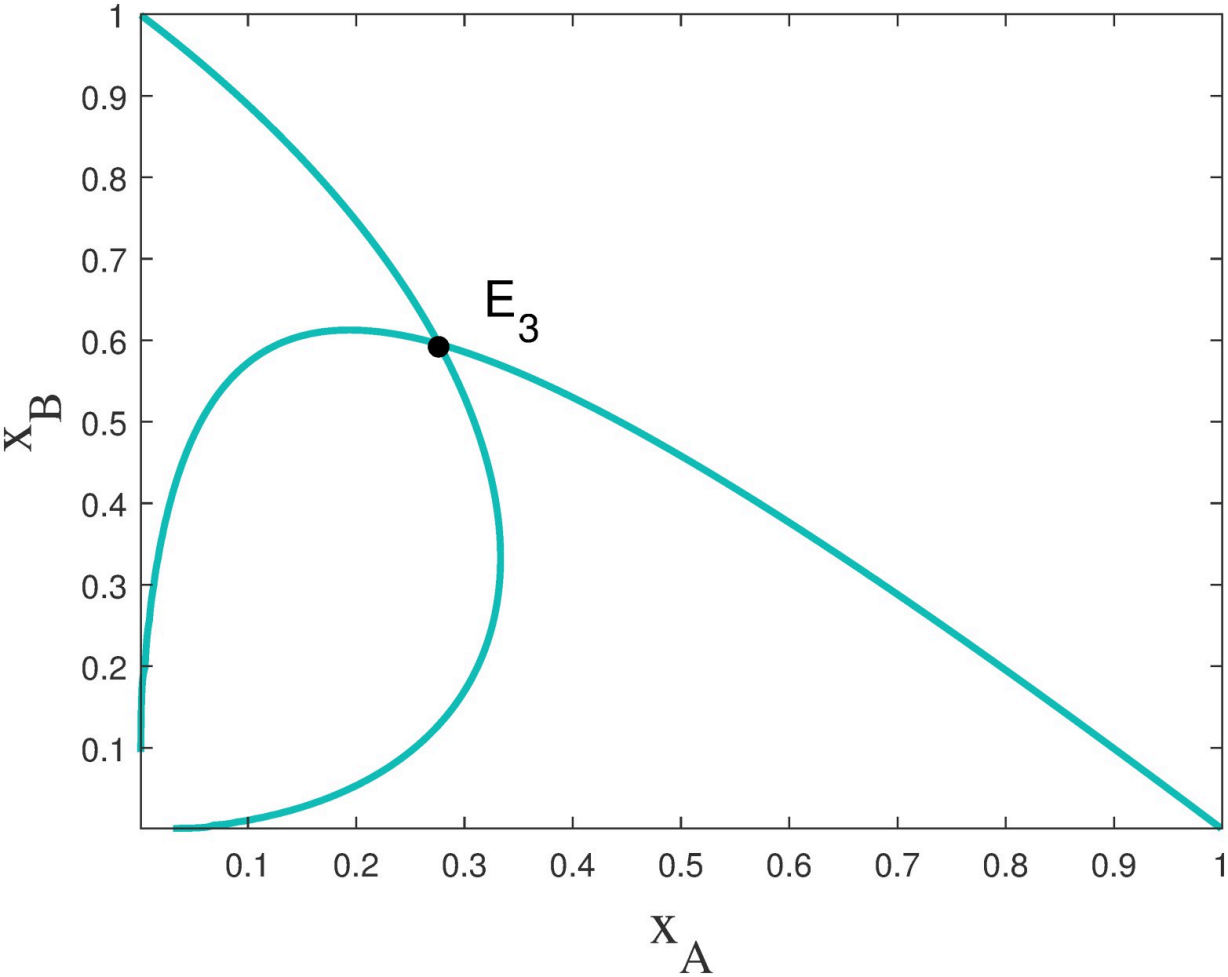
Hyperbolic nullclines for *a* = 2, *a*_1_ = 10, *a*_2_ = *a*_3_ = *a*_4_ = 1. In the figure there is a nontrivial solution $E_{3}\left(x_{A}^{*},x_{B}^{*}\right)$.

In summary, the model (4) has the nontrivial solution $E_{3}\left(x_{A}^{*},x_{B}^{*}\right)$, as shown in Fig 3.

### Stability analysis

To analyze the stability of equilibria points, we perform a classical stability analysis on equilibria points of the model (4).

For each equilibrium point of the model (4), we calculate the Jacobian matrix at that point and the corresponding *p*, *q* values to determine the stability of each equilibrium point, then the Jacobian matrix *J*, *p*, *q* values corresponding to model (4) are

$$J\left(x_{A},x_{B}\right)=\left(\begin{array}{c} \begin{array}{cc} (a_{1}a-a_{1}ax_{B})x_{A}^{a-1}-a_{1}(a+1)x_{A}^{a}-a_{2}x_{B}^{a} & -a_{1}x_{A}^{a}-a_{2}ax_{A}x_{B}^{a-1}\\ -a_{3}x_{B}^{a}-a_{4}ax_{B}x_{A}^{a-1} & (a_{3}a-a_{3}ax_{A})x_{B}^{a-1}-a_{3}(a+1)x_{B}^{a}-a_{4}x_{A}^{a} \end{array} \end{array}\right)$$ (6)

$$\begin{array}{c} \begin{array}{c} p=-[\left(a_{1}a-a_{1}ax_{B}\right)x_{A}^{a-1}-a_{1}\left(a+1\right)x_{A}^{a}-a_{2}x_{B}^{a}+\left(a_{3}a-a_{3}ax_{A}\right)x_{B}^{a-1}-\\ a_{3}\left(a+1\right)x_{B}^{a}-a_{4}x_{A}^{a}] \end{array} \end{array}$$ (7)

$$\begin{array}{c} \begin{array}{c} q=[\left(\left(a_{1}a-a_{1}ax_{B}\right)x_{A}^{a-1}-a_{1}\left(a+1\right)x_{A}^{a}-a_{2}x_{B}^{a}\right)(\left(a_{3}a-a_{3}ax_{A}\right)x_{B}^{a-1}-\\ a_{3}\left(a+1\right)x_{B}^{a}-a_{4}x_{A}^{a})-\left(-a_{1}x_{A}^{a}-a_{2}ax_{A}x_{B}^{a-1}\right)\left(-a_{3}x_{B}^{a}-a_{4}ax_{B}x_{A}^{a-1}\right)] \end{array} \end{array}$$ (8)

For the equilibrium point *E*_0_(0, 0), bringing *E*_0_(0, 0) into Eqs (6), (7) and (8). Assume *a* = 1, the Jacobian matrix is $J_{E_{0}\left(0,0\right)}=(\begin{array}{cc} a_{1} & 0\\ 0 & a_{3} \end{array}),p=-\left(a_{1}+a_{3}\right),q=a_{1}a_{3}$. Because the model parameters are set to non-negative, *p* < 0, *q* > 0, *E*_0_(0, 0) is the instability point. Assume *a* > 1, the Jacobian matrix is $J_{E_{0}\left(0,0\right)}=(\begin{array}{cc} 0 & 0\\ 0 & 0 \end{array}),p=0,q=0$. *E*_0_(0, 0) is the instability point.

For the equilibrium point *E*_1_(0, 1), bringing *E*_1_(0, 1) into Eqs (6), (7) and (8). Assume *a* = 1, the Jacobian matrix is $J_{E_{1}\left(0,1\right)}=(\begin{array}{cc} -a_{2} & 0\\ -a_{3}-a_{4} & -a_{3} \end{array})$, *p* = *a*_2_ + *a*_3_, *q* = *a*_2_*a*_3_. Assume *a* > 1, the Jacobian matrix is $J_{E_{1}\left(0,1\right)}=(\begin{array}{cc} -a_{2} & 0\\ -a_{3} & -a_{3} \end{array}),p=a_{2}+a_{3},q=a_{2}a_{3}$. Because the model parameters are set to non-negative, *p* > 0, *q* > 0, *E*_1_(0, 1) is a stable point and *p*^2^ ⩾ 4 *q*, *E*_1_(0, 1) is a node. Therefore, *E*_1_(0, 1) is a stable node. For the equilibrium point *E*_2_(1, 0), bringing *E*_2_(1, 0) into Eqs (6), (7) and (8). Assume *a* = 1, the Jacobian matrix is $J_{E_{1}\left(0,1\right)}=(\begin{array}{cc} -a_{1} & -a_{1}-a_{2}\\ 0 & -a_{4} \end{array})$. Assume *a* > 1, the Jacobian matrix is $J_{E_{1}\left(0,1\right)}=(\begin{array}{cc} -a_{1} & -a_{1}\\ 0 & -a_{4} \end{array})$, where *p* = *a*_1_ + *a*_4_, *q* = *a*_1_*a*_4_. Because the model parameters are set to non-negative, *p* > 0, *q* > 0, *E*_2_(1, 0) is a stable point, and *p*^2^ ⩾ 4 *q*, *E*_2_(1, 0) is a node. Therefore, *E*_2_(1, 0) is a stable node. The above two cases coincide with the results of model (4) study. In the bilingual competition of Reality Society, bilingual group will not last but will become monolingual group, and eventually only one language will exist. For example, in the case of the Shui nationality in Yunnan, they steadily speak only Chinese and not their own language.

For the equilibrium point $E_{3}\left(x_{A}^{*},x_{B}^{*}\right)$, bringing $E_{3}\left(x_{A}^{*},x_{B}^{*}\right)$ into Eqs (6), (7) and (8). The Jacobian matrix:

$$\begin{array}{c} \begin{array}{c} J\left(x_{A}^{*},x_{B}^{*}\right)=(\begin{array}{cc} A_{11} & A_{12}\\ A_{21} & A_{22} \end{array}) \end{array} \end{array}$$ (9)

$$\begin{array}{c} \begin{array}{c} p=-(A_{11}+A_{22}) \end{array} \end{array}$$ (10)

$$\begin{array}{c} \begin{array}{c} q=A_{11}A_{22}-A_{12}A_{21} \end{array} \end{array}$$ (11)

where $A_{11}=\left(a_{1}a-a_{1}ax_{B}^{*}\right){\left(x_{A}^{*}\right)}^{a-1}-a_{1}\left(a+1\right){\left(x_{A}^{*}\right)}^{a}-a_{2}{\left(x_{B}^{*}\right)}^{a}$,

$A_{12}=-a_{1}{\left(x_{A}^{*}\right)}^{a}-a_{2}ax_{A}^{*}{\left(x_{B}^{*}\right)}^{a-1}$,

$A_{21}=-a_{3}{\left(x_{B}^{*}\right)}^{a}-a_{4}ax_{B}^{*}{\left(x_{A}^{*}\right)}^{a-1}$,

$A_{22}=\left(a_{3}a-a_{3}ax_{A}^{*}\right){\left(x_{B}^{*}\right)}^{a-1}-a_{3}\left(a+1\right){\left(x_{B}^{*}\right)}^{a}-a_{4}{\left(x_{A}^{*}\right)}^{a}$.

Assume *a* = 1, The model (4) is written as:

$$\begin{array}{c} \left\{ \begin{array}{c} \frac{dx_{A}}{dt}=a_{1}\left(1-x_{A}-x_{B}\right)x_{A}-a_{2}x_{A}x_{B},\\ \frac{dx_{B}}{dt}=a_{3}\left(1-x_{A}-x_{B}\right)x_{B}-a_{4}x_{B}x_{A}. \end{array}\right. \end{array}$$ (12)

Computing the equilibrium point $E_{3}\left(x_{A}^{*},x_{B}^{*}\right)$, let the right-hand side of Eq (12) be zero to obtain, $x_{A}^{*}=\frac{a_{2}a_{3}}{a_{1}a_{4}+a_{2}a_{3}+a_{2}a_{4}},x_{B}^{*}=\frac{a_{1}a_{4}}{a_{1}a_{4}+a_{2}a_{3}+a_{2}a_{4}}$. Bringing $E_{3}\left(x_{A}^{*},x_{B}^{*}\right)$ into Eqs (10) and (11), $p=\frac{a_{1}a_{2}a_{3}+a_{2}a_{3}a_{4}}{a_{1}a_{4}+a_{2}a_{3}+a_{2}a_{4}},q=\frac{-a_{1}a_{2}a_{3}a_{4}}{a_{1}a_{4}+a_{2}a_{3}+a_{2}a_{4}}$. Assume *a* > 1, *q* = *A*_11_*A*_22_ − *A*_12_*A*_21_ < 0. Because the model parameters are set to non-negative *a*_1_ > 0, *a*_2_ > 0, *a*_3_ > 0, *a*_4_ > 0, *q* < 0. $E_{3}\left(x_{A}^{*},x_{B}^{*}\right)$ is the instability point.

**Theorem 2**. *Assume a* ⩾ 1, *a*_1_ > 0, *a*_2_ > 0, *a*_3_ > 0, *a*_4_ > 0, *we have E*_1_(0, 1), *E*_2_(1, 0) *are stable nodes*. $E_{0}\left(0,0\right),E_{3}\left(x_{A}^{*},x_{B}^{*}\right)$ *are instability point*.

## Numerical simulation

In this section, we perform numerical simulations for model (3). We divide this section into five examples. Example 1 illustrates the case of stable node *E*_1_, Example 2 is the case of stable node *E*_2_, Example 3 illustrates the effect of parameter $s_{x_{A}}$, Example 4 introduces the role of parameter $I_{w\cdot x_{A}}$, and Example 5 studies the effect of the initial distribution on the bilingual competitive model. We will analyze the different cases of the set of parameter values $\left\{s_{x_{A}},s_{x_{B}},P_{w\cdot x_{A}},P_{w\cdot x_{B}},x_{A},x_{B}\right\}$ of in the bilingual competitive model without sliding mode control. In this paper, all numerical solutions of the set of differential equations are obtained and visualized using MATLAB software. We set to *μ* = 0.02, because losing a language through generations is a much slower process than learning another language in the course of an individual’s life.

In example 1, we will see a situation where the only persistent monolingual group. The model parameters are shown in Table 1.

**Table 1:** The case of stable node *E*_1_.

parameter	value	parameter	value	initial	value
$s_{x_{A}}$	0.4	$I_{w\cdot x_{B}}$	1	*x*_*A*_(0)	0.33
$s_{x_{B}}$	0.6	$I_{x_{A}\cdot w}$	1	*x*_*B*_(0)	0.33
$I_{w\cdot x_{A}}$	10	$I_{x_{B}\cdot w}$	1	*w*(0)	0.34

**Fig 4:**
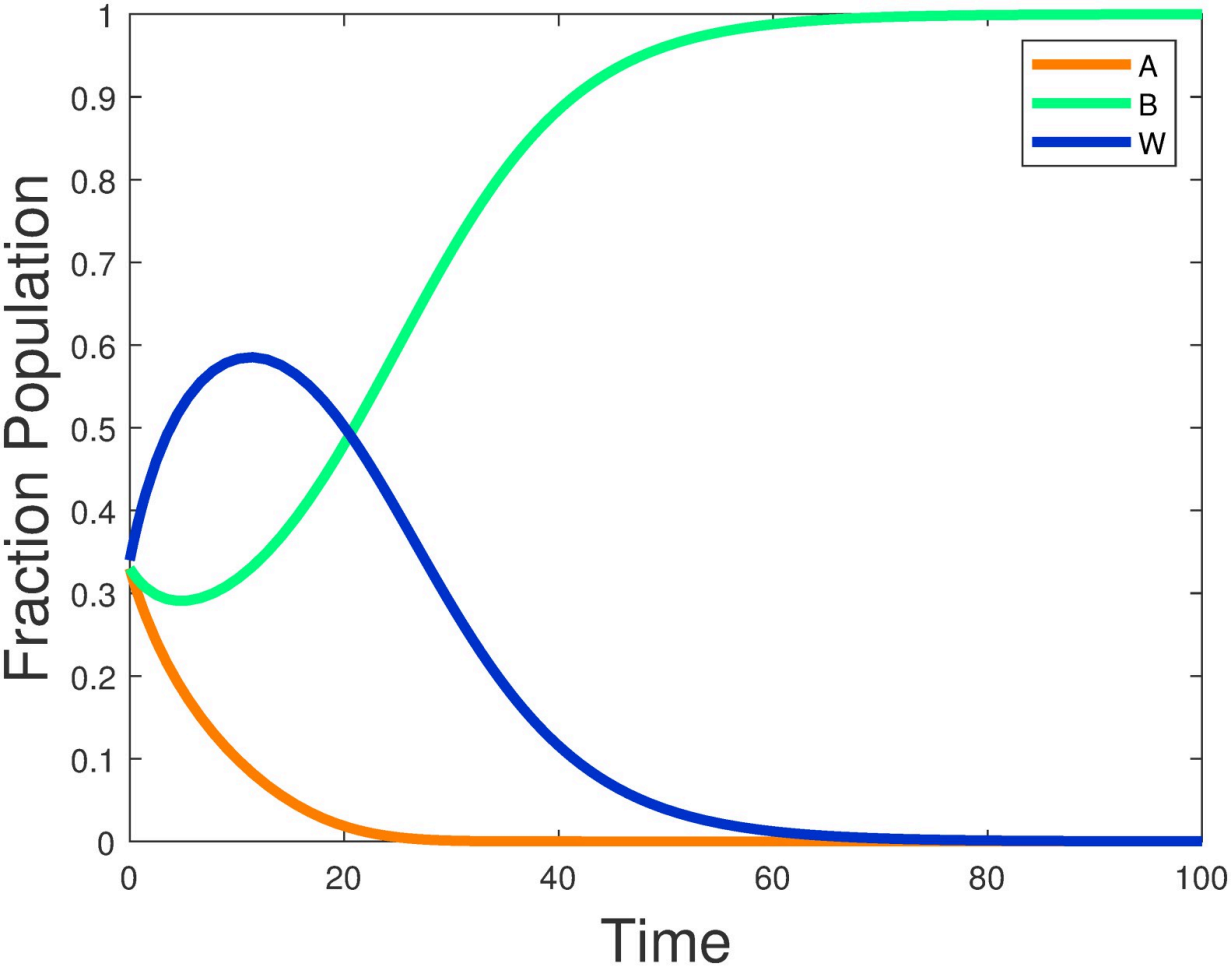
Stable node (0, 1, 0) of model (3) without sliding control.

In this example the parameters are set as shown above: the value of parameter *a* is 1.31, the status of language *A*, *B* is 0.4, 0.6 respectively. Compared to language *A*, language *B* has a higher status in the society and its economic benefits are better. The parameter $I_{x_{A}\cdot w},I_{x_{B}\cdot w},I_{w\cdot x_{B}}$ is set to 1, which indicates the intercommunication from adult life from monolingual *A*(*B*) to bilinguals or bilinguals to monolingual *B*. With $I_{w\cdot x_{A}}$ set to 10, the interaction value is higher because of hobbies or other reasons and people are more willing to learn the language A. The value of $I_{x_{A}\cdot w},I_{x_{B}\cdot w}$ is lower compared to $I_{w\cdot x_{A}}$ because learning a new language as an adult is not as easy as it was as a child. This parameter indicates that the number of speakers increases linearly with language status. When the initial distribution parameter *x*_*A*_(0), *x*_*B*_(0) is 0.33, over time, satisfies the equilibrium point and stabilizes, as shown in Fig 4. Eventually, people will only speak language B, and language will die out, which is consistent with the theory in Section 3.

In example 2, we will see a situation where the only persistent monolingual group *A*. The model parameters are shown in Table 2.

**Table 2:** The case of stable node *E*_2_.

parameter	value	parameter	value	initial	value
$s_{x_{A}}$	0.6	$I_{w\cdot x_{B}}$	1	*x*_*A*_(0)	0.33
$s_{x_{B}}$	0.4	$I_{x_{A}\cdot w}$	1	*x*_*B*_(0)	0.33
$I_{w\cdot x_{A}}$	10	$I_{x_{B}\cdot w}$	1	*w*(0)	0.34

**Fig 5:**
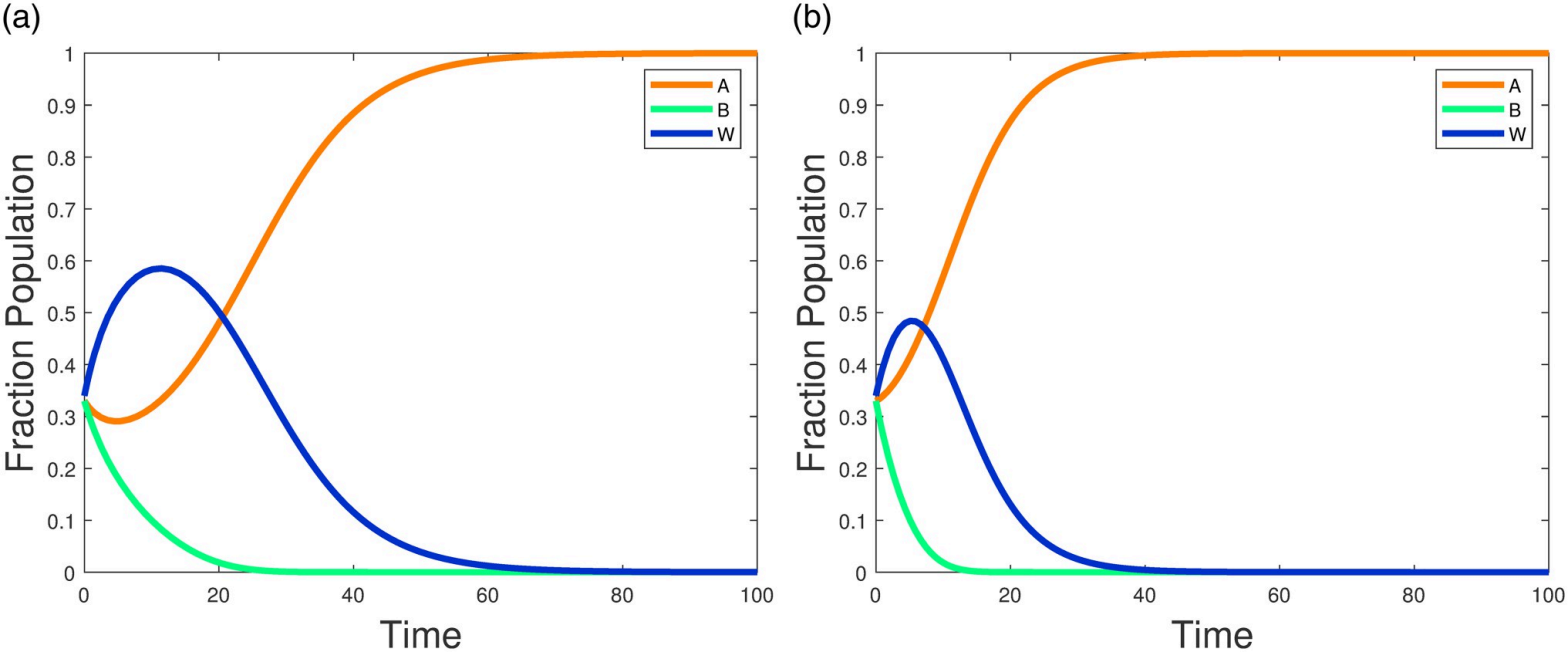
Stable node (0, 1, 0) of model (3) without sliding control. (a)$s_{x_{A}}=0.6$. (b)$s_{x_{A}}=0.9$.

In this example, the parameters are set as shown in Table 2. Unlike in example 1, the status of language *A* is higher, the interaction value from bilingualism to language *A* is higher, and people are more willing to learn language *A* because of the greater economic effect of the higher status of language *A*. Over time, the equilibrium point *E*_2_ is satisfied and stabilizes, as shown in Fig 5(a) below. People will only speak language *A* and language *B* will die out, which is consistent with the theory in Section 3. To investigate the effect of language status on language evolution, the parameters are set to $s_{x_{A}}=0.9,s_{x_{B}}=0.1$. Eventually, the stable node *E*_2_ is satisfied and all individuals in the community will only speak language *A*, and language *B* will die out. Compared with Fig 5(a) and 5(b) accelerates the extinction of language *B*, and the language *B* community dies out in a shorter period. Therefore, language extinction can be slowed down by changing the status of the language. Changing the status of a language is a good policy in the short time to maintain the bilingual status of the language.

In example 2 the effect of language status on language evolution is illustrated, but not specifically how. In Example 3 below we present the effect of language status on bilingual competitive groups.

**Fig 6:**
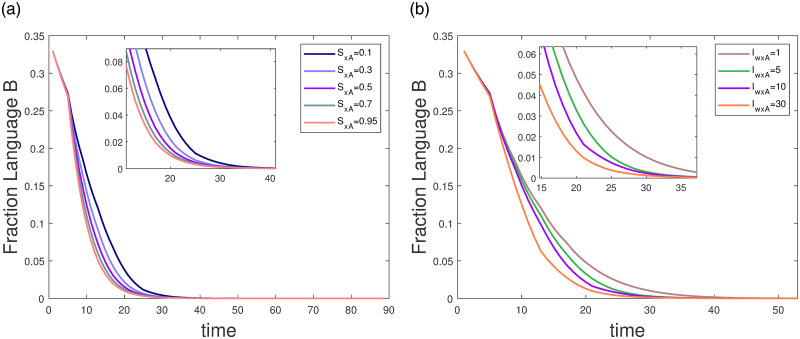
The effect of parameter (a)$s_{x_{A}}$; (b)$I_{w\cdot x_{A}}$.

In example 3, the parameters are set to $s_{x_{A}}=\left\{0.1,0.3,0.5,0.7,0.95\right\}$ $I_{w\cdot x_{A}}=10,I_{w\cdot x_{B}}=1,I_{x_{A}\cdot w}=1,I_{x_{B}\cdot w}=1,x_{A}\left(0\right)=0.33,x_{B}\left(0\right)=0.33,w\left(0\right)=0.33,,s_{x_{B}}=1-s_{x_{A}}$, as shown in Fig 6(a). The vertical coordinate in Fig 6(a) is the population percentage of language *B* in Fig 5. Fig 6(a) shows that as $s_{x_{A}}$ increases, the rate of population percentage extinction of language *B* increases, accelerating the extinction of language *B* in a short period. The disappearance of languages can be effectively slowed down by changing the parameter $s_{x_{A}}$.

In example 4, we discuss the effect of parameter $I_{w\cdot x_{A}}$. The parameter are set to $s_{x_{A}}=0.6,s_{x_{B}}=0.4,I_{x_{A}\cdot w}=I_{x_{B}\cdot w}=I_{w\cdot x_{B}}=1,x_{A}\left(0\right)=0.33,x_{B}\left(0\right)=0.33,w\left(0\right)=0.34,I_{w\cdot x_{A}}=\left\{1,5,10,30\right\}$, as shown in Fig 6(b). As $I_{w\cdot x_{A}}$ increases, the extinction of language *B* becomes faster, indicating that decreasing the interaction value from bilingual to monolingual *A* slows down the disappearance of language *B*.

**Fig 7:**
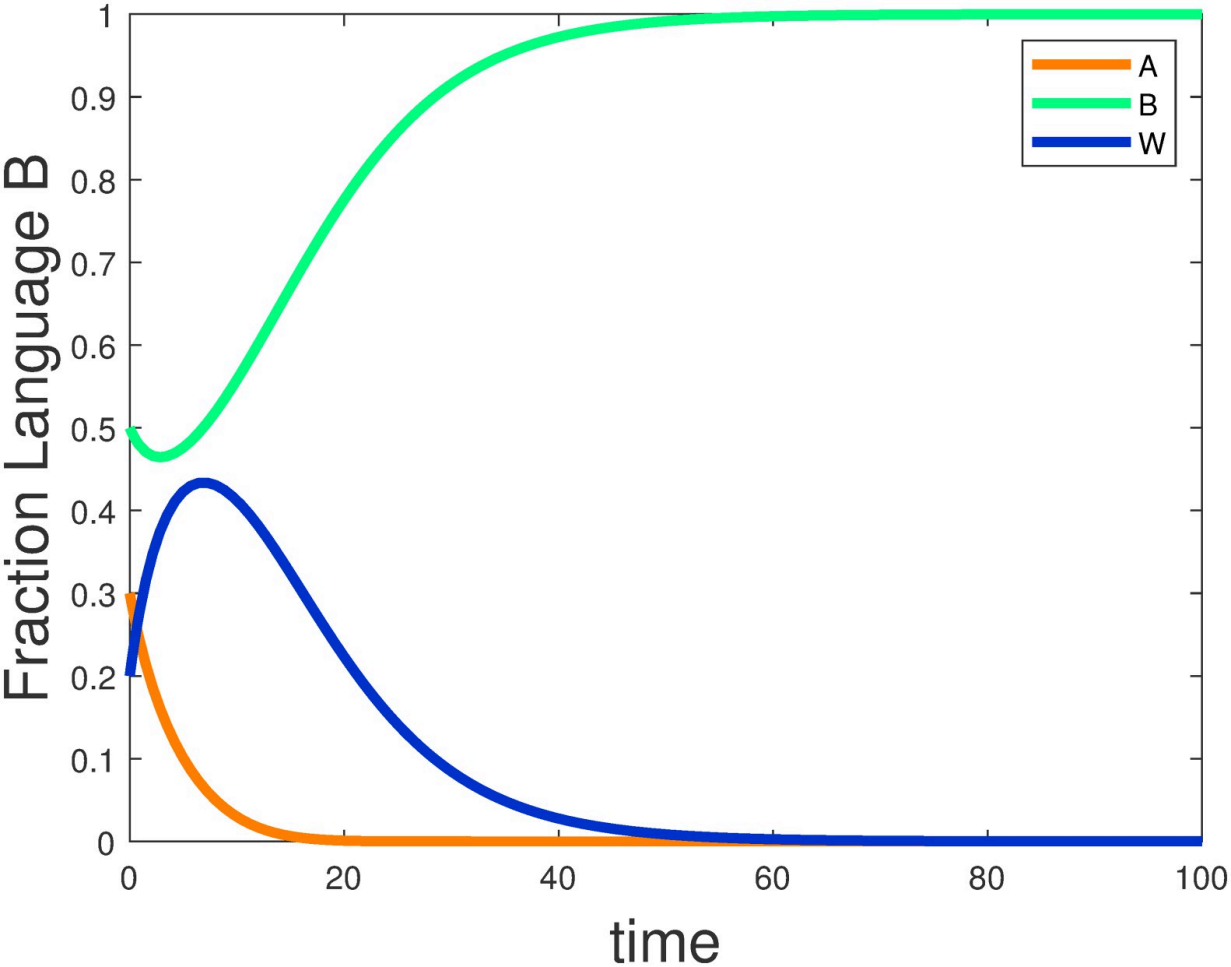
The effect of the initial distribution.

In example 5, we study the effect of the initial distribution of languages on the evolution of languages. The parameter are set to $s_{x_{A}}=0.4,s_{x_{B}}=0.6,I_{w\cdot x_{A}}=10,I_{w\cdot x_{B}}=I_{x_{A}\cdot w}=I_{x_{B}\cdot w}=1$, the initial distribution is set to *x*_*A*_(0) = 0.3, *x*_*B*_(0) = 0.5, indicating that the language groups *A*, *B* within the community are not uniformly distributed and appear heterogeneous. Over time, the equilibrium point *E*_1_ is satisfied and stabilizes, as shown in Fig 7. People will only speak language *A* and language *B* will die out. Compared to Fig 4, the rate of extinction is faster in Fig 7. To make languages coexist, the initial distribution of languages can be adjusted appropriately, but the initial distribution is influenced by geographical factors and is less mobile and not easily changed.

In summary, to slow down the rate of language extinction, the government could introduce policies to raise the status of endangered languages and increase interaction and increase the value of interaction from bilingualism to monolingualism, increasing the population of endangered languages. However, the initial distribution is influenced by geographical factors that do not change.

## Bilingual competition model with sliding mode control

To achieve language coexistence, when a language is identified as endangered, some policies should be implemented to achieve language coexistence. To provide some theoretical support for policy designation, this paper proposes a quantitative model that makes language coexistence possible when languages face endangerment through a bilingual competition model with sliding mode control.

Bilingual competition model with sliding mode control:

$$\begin{array}{c} \left\{ \begin{array}{c} \frac{dx_{A}}{dt}=\mu wI_{w\cdot x_{A}}s_{x_{A}}x_{A}^{a}-\left(1-\mu \right)x_{A}I_{x_{A}\cdot w}s_{x_{B}}x_{B}^{a},\;x_{B}⩾ET\\ \frac{dx_{B}}{dt}=\mu wI_{w\cdot x_{B}}s_{x_{B}}x_{B}^{a}-\left(1-\mu \right)x_{B}I_{x_{B}\cdot w}s_{x_{A}}x_{A}^{a}.\;x_{B}⩾ET\\ \frac{dx_{A}}{dt}=\mu w{\hat{I}}_{w\cdot x_{A}}{\hat{s}}_{x_{A}}x_{A}^{a}-\left(1-\mu \right)x_{A}{\hat{I}}_{x_{A}\cdot w}{\hat{s}}_{x_{B}}x_{B}^{a},\;x_{B}<ET\\ \frac{dx_{B}}{dt}=\mu w{\hat{I}}_{w\cdot x_{B}}{\hat{s}}_{x_{B}}x_{B}^{a}-\left(1-\mu \right)x_{B}{\hat{I}}_{x_{B}\cdot w}{\hat{s}}_{x_{A}}x_{A}^{a}.\;x_{B}<ET \end{array}\right. \end{array}$$ (13)

The parameter $x_{A},x_{B},w,\mu ,I_{x_{A}\cdot w},I_{x_{B}\cdot w},s_{x_{A}},s_{x_{B}}$ has the same meaning as Eq (3). ${\hat{I}}_{x_{A}\cdot w},{\hat{I}}_{x_{B}\cdot w}$ denotes the value of interaction from monolingual *A*, *B* to bilingual individuals after sliding mode control, and parameter ${\hat{s}}_{x_{A}},{\hat{s}}_{x_{A}}$ denotes the status of language *A*, *B* after sliding mode control, respectively. *ET* stands for Endangerment Threshold.

We assume that the models are valid before and after the sliding mode control. Table 3 shows the parameter settings of the bilingual competitive model without sliding mode control; when *ET* = 0.3, *x*_*B*_ < 0.3, sliding mode control is applied to the bilingual competitive model, the parameters are set to Tables 4–6 below, the value of parameter *a* is 1.

**Table 3:** Without sliding mode control.

parameter	value	parameter	value	initial	value
$s_{x_{A}}$	0.8	$I_{w\cdot x_{A}}$	1	$I_{x_{A}\cdot w}$	0.03
$s_{x_{B}}$	0.2	$I_{w\cdot x_{B}}$	1	$I_{x_{B}\cdot w}$	0.03

**Table 4:** Sliding mode control language status.

parameter	value	parameter	value	initial	value
$s_{x_{A}}$	0.6	$I_{w\cdot x_{A}}$	1	$I_{x_{A}\cdot w}$	0.03
$s_{x_{B}}$	0.4	$I_{w\cdot x_{B}}$	1	$I_{x_{B}\cdot w}$	0.03

**Table 5:** Sliding mode control interaction values.

parameter	value	parameter	value	initial	value
$s_{x_{A}}$	0.8	$I_{w\cdot x_{A}}$	1	$I_{x_{A}\cdot w}$	0.06
$s_{x_{B}}$	0.2	$I_{w\cdot x_{B}}$	1	$I_{x_{B}\cdot w}$	0.06

**Table 6:** Sliding mode control language status and interaction values.

parameter	value	parameter	value	initial	value
$s_{x_{A}}$	0.6	$I_{w\cdot x_{A}}$	1	$I_{x_{A}\cdot w}$	0.06
$s_{x_{B}}$	0.4	$I_{w\cdot x_{B}}$	1	$I_{x_{B}\cdot w}$	0.06

**Fig 8:**
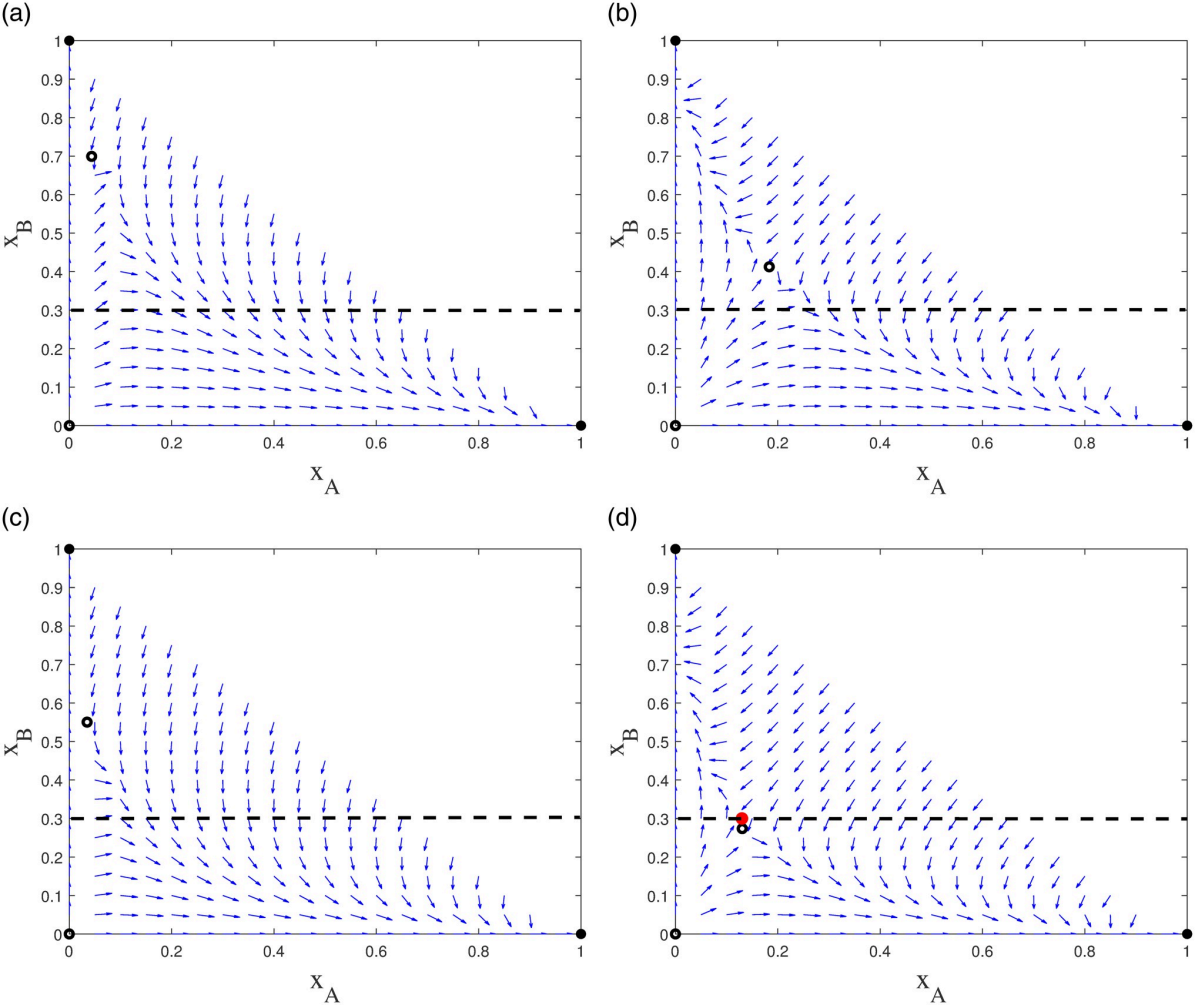
Comparison of the impact of different strategies for bilingual competition model with sliding mode control. The black solid point represents the stable point, the black empty point is the unstable point. (a) Flow diagrams for the dynamics of model (3) without sliding mode control; (b) Flow diagrams for the dynamics of model (3) with sliding mode control, from $s_{x_{B}}=0.2$ to $s_{x_{B}}=0.4$ when *x*_*B*_ < 0.3; (c) Flow diagrams for the dynamics of model (3) with sliding mode control, from $I_{x_{A}\cdot w}=0.03,I_{x_{B}\cdot w}=0.03$ to $I_{x_{A}\cdot w}=0.06,I_{x_{B}\cdot w}=0.06$ when *x*_*B*_ < 0.3; (d) Flow diagrams for the dynamics of model (3) with sliding mode control, from $s_{x_{B}}=0.2,I_{x_{A}\cdot w}=0.03,I_{x_{B}\cdot w}=0.03$ to $s_{x_{B}}=0.4,I_{x_{A}\cdot w}=0.06,I_{x_{B}\cdot w}=0.06$ when *x*_*B*_ < 0.3.

The direction fields of the bilingual competitive model without sliding mode control are shown in Fig 8(a). The four equilibrium points of the model are *E*_0_(0, 0), *E*_1_(0, 1), *E*_2_(1, 0), *E*_3_(0.0437, 0.6993). The black solid point represents the stable point *E*_1_, *E*_2_, and the black empty point is the unstable point *E*_0_, *E*_3_. And *E*_3_(0.0437, 0.6993) is an unstable point, so the coexistence of languages cannot be achieved. When *x*_*B*_ < 0.3, almost all trajectories converge to *E*_2_(1, 0), then language *B* dies. To make the languages coexist, the instability point *E*_3_(0.1833, 0.4125) is introduced by elevating the status of language *B* from $s_{x_{B}}=0.2$ to $s_{x_{B}}=0.4$ when *x*_*B*_ < 0.3 in Fig 8(b). However, the intervention policy to raise the status of language *B* from 0.2 to 0.4 does not achieve language coexistence.

By increasing the value of interaction between adults from monolingual *A*, *B* to bilingual individuals, from $I_{x_{A}\cdot w}=0.03,I_{x_{B}\cdot w}=0.03$ to $I_{x_{A}\cdot w}=0.06,I_{x_{B}\cdot w}=0.06$, introducing the instability point *E*_3_(0.0348, 0.5503), also the coexistence of languages is not achieved. As shown in Fig 8(c), when *x*_*B*_ < 0.3, the direction of the vector field will finally reach the stability point *E*_2_(1, 0), language B dies. Therefore, raising the value of monolingual to bilingual interaction from 0.03 to 0.06 does not achieve linguistic coexistence.

Also raising the status of language *B* and increasing the value of the interaction from monolingual to bilingual, from $s_{x_{B}}=0.2,I_{x_{A}\cdot w}=0.03,I_{x_{B}\cdot w}=0.03$ to $s_{x_{B}}=0.4,I_{x_{A}\cdot w}=0.06,I_{x_{B}\cdot w}=0.06$, introduces *E*_3_(0.1305, 0.2937). So that when *x*_*B*_ < 0.3, the direction of the vector field goes up; when *x*_*B*_ > 0.3, the direction of the vector field goes down. As shown in Fig 8(d), this allows the introduction of the pseudo-equilibrium point s*(0.13, 0.3, 0.57), where the track on the upper side of the point goes down and the track on the lower side goes up. Thus, the possibility of language coexistence is increased by raising the status and interaction value of languages.

## Conclusion

We perform a qualitative theoretical analysis of the bilingual competitive model without sliding mode control, the trivial and non-trivial solutions of the model is obtained, and the solution of the model is proved to have positive invariance. Using the classical stability analysis, the stability of the trivial and non-trivial solutions is obtained. In addition, to keep the language diversity and make language coexist, this paper proposes a new bilingual competition model with sliding mode control, which introduces pseudo-equilibrium points by proposing sliding mode control to the model, thus improving the possibility of language coexistence. Meanwhile, numerical simulations clearly illustrate the effectiveness of the sliding mode control strategy. In the community, the government can prevent the mass extinction of languages by introducing policies to make the status of languages higher, such as giving financial support and establishing bilingual schools; the government can also encourage monolinguals to learn other languages and promote monolingual-to-bilingual interaction.

However, due to the lack of data on ethnic minorities in Yunnan Province, the data were not fitted and analyzed, and the comparative verification of real data should be considered later. Since the model only depicts the existence of bilingualism in the community, while the actual situation may have three or more languages, such as in Xishuangbanna, a border area in Yunnan Province, where they know Dai and Lahu languages and also Chinese. The model should be extended to three languages, etc. This paper proposes to adjust the status and interaction values of languages to make language coexistence more likely, the next possible research is in which range of parameters is easier to achieve coexistence?

## Supporting information

S1 File (ZIP)
